# Supplementary figures and images for: Normative assembly rule reveals fairness in microbial communities
Source: PLoS Biol. 2026 Jun 24;24(6):e3003872. doi: 10.1371/journal.pbio.3003872 (PMC13327520; doi:10.1371/journal.pbio.3003872)

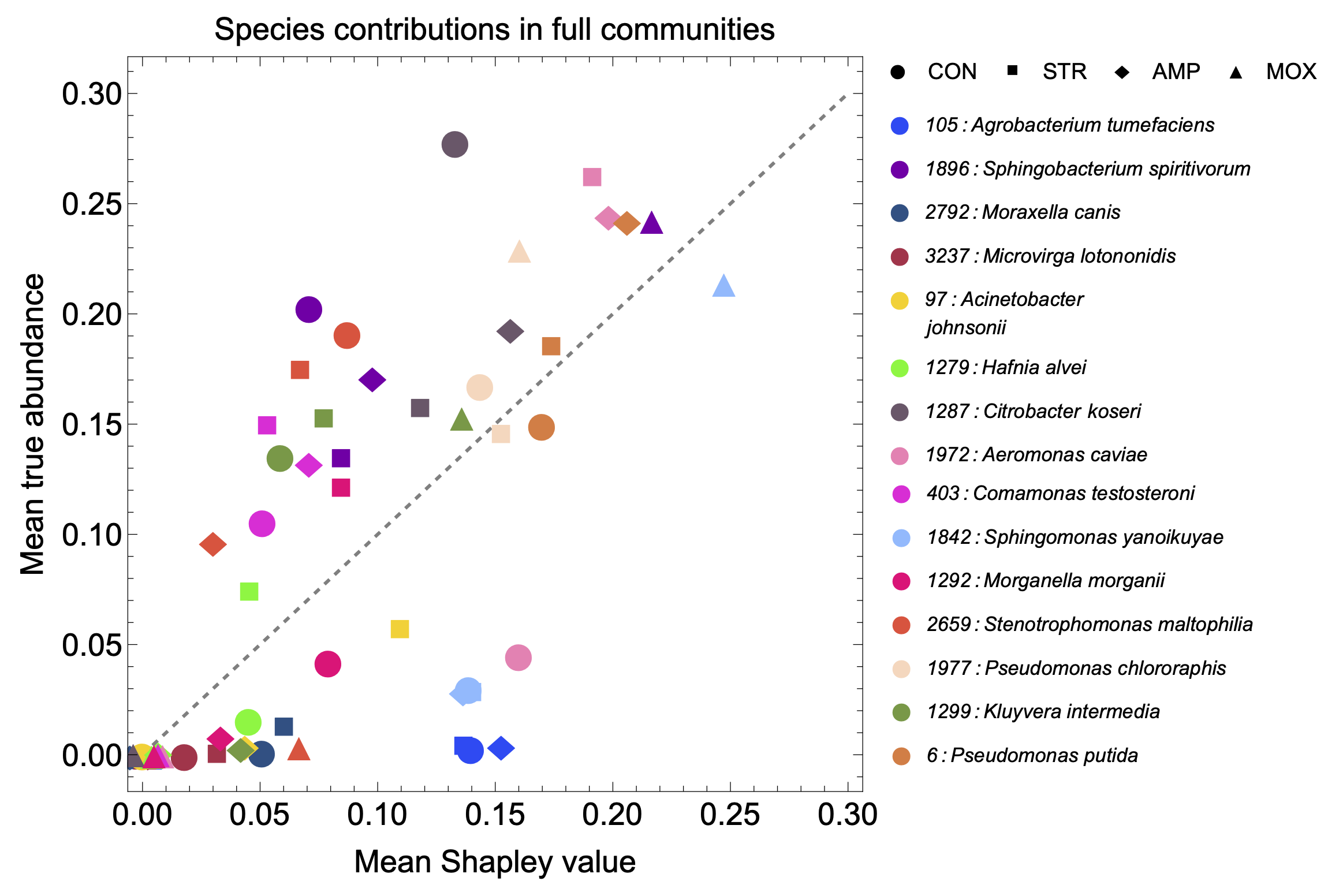

Supplement: S1 Fig — The Shapley values of each species (given by the color label) was averaged over all the communities it was present and plotted separately for each environment (given by the plot marker) against the corresponding mean true abundance. Fig 2D is obtained from this plot by further averaging these points across the environments per species. (TIFF) [file pbio.3003872.s001.tiff]

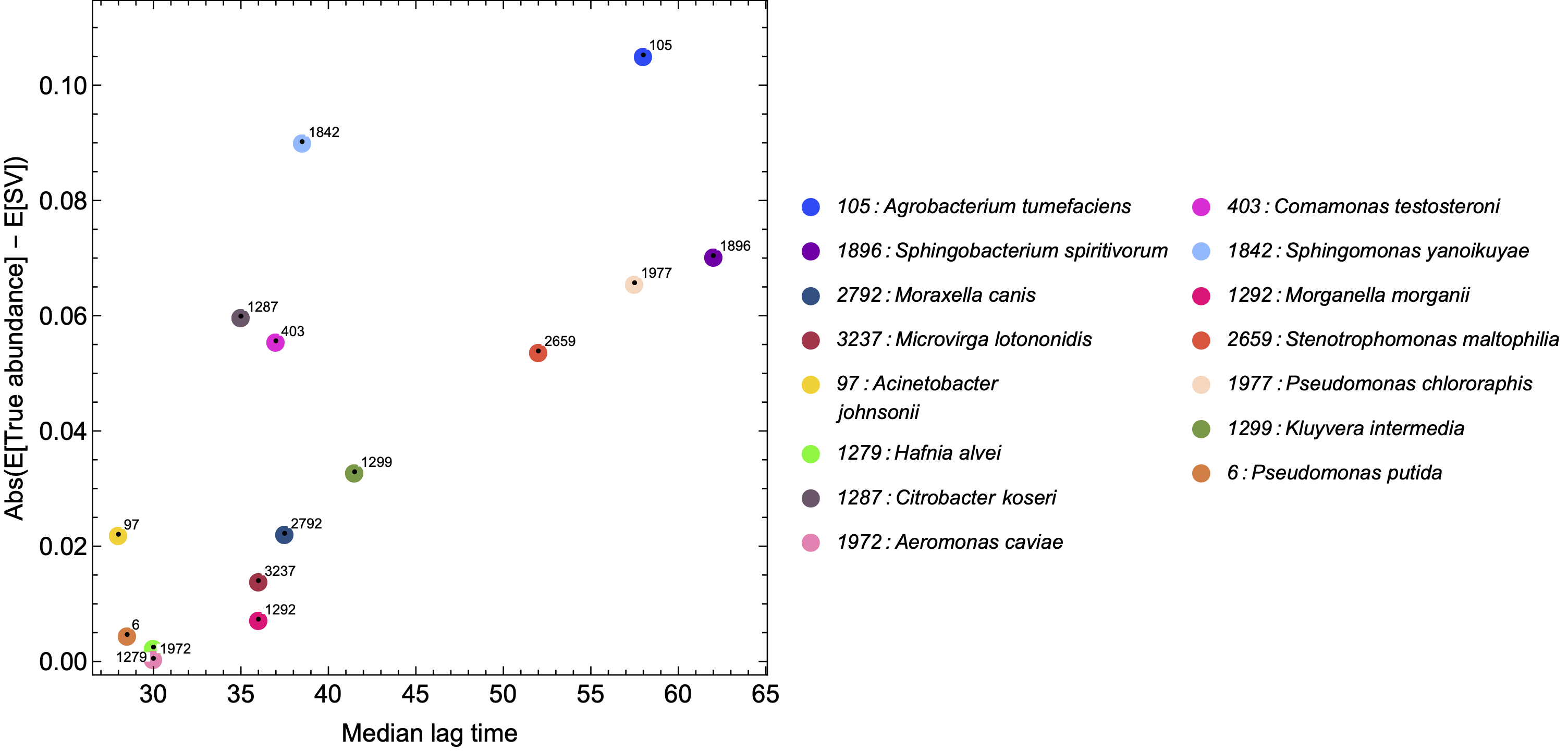

Supplement: S2 Fig — Using the optical density measurements, we first computed the lag time associated with each growth curve as the time until maximum growth rate. We then plotted the median lag time of each species against the observed unfairness (absolute distance to the diagonal in Fig 2D). This shows that species with shortest lag time (that is, those that start to grow earlier) are also those which on average obtain their fair share according to their Shapley value, while species with longer lag time reach either unfairly high or low abundances. (TIFF) [file pbio.3003872.s002.tiff]
