# Supplementary material for: Normative assembly rule reveals fairness in microbial communities
Source: PLoS Biol. 2026 Jun 24;24(6):e3003872. doi: 10.1371/journal.pbio.3003872 (PMC13327520; doi:10.1371/journal.pbio.3003872)
Supplement: Attachment — Submitted filename: Response-Letter.pdf [file pbio.3003872.s003.pdf]

RESPONSE TO REVIEWERS' COMMENTS

Dear Editor,

We'd like to thank the Reviewers and Editor(s) for the encouraging and helpful comments. We have implemented a series of modifications throughout the manuscript based on the received feedback and provide a detailed response case-by-case below. We believe we have addressed all points constructively.

Sincerely,
Teemu Kuosmanen and Ville Mustonen
(on behalf of all Authors)

Reviewer #1:

The manuscript "Normative assembly rule reveals fairness in microbial communities" introduces the idea of Shapley values to community ecologists and uses microbial growth assays as a first case study. The work is presented clearly, the limitations (e.g., need to assemble all possible combinations of species) are explicitly addressed, and exciting future directions are proposed. It is an excellent example of the value of cross-disciplinary research, and microbial systems are the perfect starting point for introducing these ideas to ecology. I was not familiar with the concept of Shapley values before, and this manuscript made me think deeply about the trade-offs between the mechanistic and phenomenological approaches ecologists take when modelling community assembly.

We'd like to thank Reviewer 1 for the nice and encouraging comments towards our work. Below we reply to the raised comments and observations case-by-case.

Based on the paragraph starting at L322, the authors present the two key applications of their work as: (i) a new model-agnostic framework for making causal inferences about species composition and interactions, and (ii) a new approach for predicting community assembly outcomes. I was very interested by the first application, but I was a little less convinced by the second.

(i) I was intrigued by how this perspective of fairness could provide new insights into the biology of community assembly. Unravelling the mechanisms that make communities fair or unfair seems like an exciting line of research. Figure S1 and the related discussion of lag time really sparked my interest as it felt intuitive how increased lag time may prevent a species from being able to use up its fair share of resources. However, it seems that increased lag time can cause a species to be both above and below the 1:1 line in Figure 2D; lags can be advantageous or disadvantageous depending on the context. How could this be happening?

Indeed, when the species niches overlap, we'd expect that increased lag would lead to unfairness, as the early growth of the other competing species limits the growth of the later species, even if that species would be more efficient. However, it is also common in microbial communities that some species may produce metabolites that only some other species may be able to utilize and which are thus not present initially. Such cross-feeding is known to play an important role and can provide one explanation why increased lag may actually be beneficial in some contexts.

We added the following sentence to discussion:

This observation may be explained by the varying degree of niche overlap, where the growth of the species with longer lag can be hindered in case of high overlap, but improved in case of low overlap, for example, due to cross-feeding.

Another striking result, where abundant species seem to always gain more biomass than their fair share (L133, Figure 2, and the hour-glass pattern in Figure 4) also intrigued me, but I thought the underlying biological mechanism could be explored a bit more. Once a species has an advantage it somehow is able to make the most of it?

Yes, the general pattern that we observe (as in Fig. 2C) is that biomass is more concentrated to the most prevalent species compared to the fair distribution. However, it is worth to note that in Figure 2 analysis, these may or may not be the same species as we first focused on the distributional properties before moving to species-specific analysis as presented in Figure 3.

To us, this empirical finding simply represents an outcome of ecological dynamics, and our goal here has been to view it as an interesting macroecological pattern that is worth reporting rather than a specific instance of a microbiological phenomenon that we wish to carefully dissect. Ultimately, we think this boils down to analyzing determinants of growth and ecological interactions, which requires additional experiments (this relates to the micro- vs. macro-level that we raised already in the introduction).

Note, however, that this need not imply “rich get richer” type of phenomenon because we are still only observing a static distribution, not how it evolves with time. Future studies could investigate both the generality of this pattern as well as its dynamics in addition to mechanisms.

Are the authors confident that this result isn't a bias associated with how the sequencing data is converted to relative frequencies?

We are confident that the observed pattern in Figure 2 is real. First, putative bias from conversion of abundances to frequencies is unlikely given that the overall variability of the yield is modest. Figure R1 shows that when we sort the experiments by their yields and select three communities with the highest yields (top row) and three communities with lowest (non zero) yields (bottom row), the pattern of Figure 2 remains qualitatively unchanged: e.g. the rank I fair yield share (Shapley) is substantially smaller than the observed rank I yields share. We further note that Figure 2 considers distribution properties of Shapley vs. Observed and does not allow addressing species identity related questions including whether or not the rank I Shapley value species is also the rank I in Observed shares. This analysis is presented in Figure 3 where we highlight different cases: e.g. in 3B the leading ranks are not the same in several conditions (strong unfairness) whereas in 3C we have a much fairer community. Second, our sequencing libraries have been carefully constructed so that reads are mapping to their respective species (cross talk minimised, see ***Library Preparation***), and we further factor in species specific 16S gene copynumber to produce high-quality species frequency data (see ***Bioinformatics/ Downstream Sequence Analysis***).

Figure R1

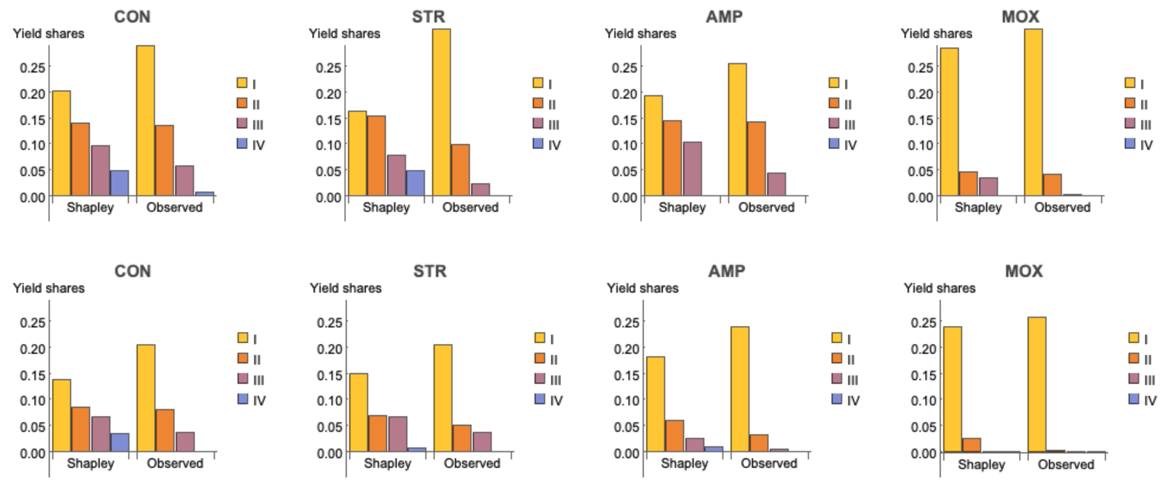

Finally, the tentative conclusion arising from Figure 3E - that fairness seems to set an upper limit to evenness - felt like it deserved much more mechanistic discussion. In general, I thought the authors could lean more into the causal inferences/biological mechanisms revealed by their fairness approach, so that it was clearer how this new way of analysing ecological data can provide new insights.

We agree that there are many interesting follow-ups that could be taken. Indeed, the value of our approach lies precisely in the fact that it can help identify such interesting patterns from the data. However, this alone won't be sufficient to get into the mechanisms underlying such patterns, for that one needs to perform additional measurements. In our view, we identified some probable mechanisms that may be at play:

In reality, the true end-frequencies after the community growth can deviate from the fair distribution because, in a competitive setting, resource efficiency (monoculture yield) is not the only important factor: for example, shorter lag time, higher resource uptake rate, consumption of secondary metabolites, exploitation of other public goods without reciprocal contribution to their production (cheating), and direct negative interactions, such as bacterial warfare (29), can all significantly change the competitive dynamics such that the monoculture yield alone is not predictive enough for success in a community setting.

We also provided some tentative analysis especially on the role of lag time in Fig. S2. As for the case of the mechanistic underpinnings of diversity, trying to address this is perhaps even more complicated, because we view diversity as a function of the underlying assembly process. In general, in our view very few studies investigate diversity as a mechanistic phenomenon by itself, but rather as a feature of data to which our study also provides some empirical insights.

We feel that digging deeper to such questions, although interesting, is out of scope for the present Short Report and wouldn't substantially add to our current discussion.

(ii) The second application of the framework - predicting community outcomes - was a bit less compelling in my opinion. Apologies if I am misunderstanding the analyses, but to me it seemed that the predictions based purely on Shapley values are shown in Figure 4A. These predictions require knowing the total yield of every combination of species, but their accuracy seems very poor considering how much data they are based on. If prediction is the goal, I doubt that the fair distribution is a good choice (the authors do rightly present it as more of a sort of null model). Figure 4B and 4C look better (still not great though given that the predicted frequencies can still be off by as much as ~0.4), but they are augmented with even more data: relative frequencies for all pairs and all triplets. Once the frequency of every species in every subcommunity can be used in the prediction (as in Figure 4C), then presumably the fair assembly rule will be outperformed by even simple statistical models, and perhaps even by minimal ecological models like a generalized Lotka-Volterra model with higher-order interactions? Unless the authors can show that their fairness framework provides better predictions than current approaches, maybe it would be better to focus more on the causal inference side of the work?

We want to emphasize here that we are not *fitting* any model to the data, so the amount of "training data" is irrelevant because there is essentially no training data or free parameters that would need to be inferred. The fair distribution given by the Shapley values provides a mathematically unique, normative baseline to which we can compare the reality. We find it very interesting that even though there is no *a priori* reason to expect this to match the reality, it nevertheless does so at least in some communities, very well (see for example communities 10 and 11 in Fig. 3A). Furthermore, in Figures 4B/C we show how unfairness can also be understood and explained in the same Shapley framework, meaning how the information about realized outcomes at lower levels can be included to "update" the prediction. This result is also not really driven by giving more data, but rather different type of data that is then used as the correct initial condition for the recursion. For example, in Figure 4B, while we use the pairwise frequency data, we don't use the yield data from monocultures, and in Figure 4C, we use the frequencies of the triplets, but not any data from lower subcommunities.

While we agree that the basic Shapley version may be too crude for predictive purposes, we still insist that reporting also these findings is valuable because it may open up totally new ways to think about community assembly and indeed predict it from first principles. This is because the normative approach advocated here is completely different from fitting simple mathematical or statistical models that require inferring various parameters. One of the main weaknesses of the Lotka-Volterra approach is that while the data can usually be fitted very well, the inferred parameters typically do not predict other outcomes in different assemblages, because the parameters themselves depend on the community context (see e.g. https://onlinelibrary.wiley.com/doi/full/10.1111/ele.70027, https://link.springer.com/article/10.1007/s12080-018-0377-0). Also, Lotka-Volterra model, especially with higher order parameters, is not in our view "minimal" in terms of model complexity as for model selection one should also somehow penalize on the number of free parameters and contrast that to our approach of zero free parameters which still provides a very parsimonious description of the observed data. As

there are many different models and ways to do this, we don’t think that the present discussion would significantly benefit from a quantitative comparison.

However, based on this comment, we have now attempted to further clarify this aspect of our work in the Discussion section more clearly:

To emphasize, we did not fit any mathematical or statistical model to data but rather derived the predictions from the basic principles of fair distribution, further showing that the recursive logic of the Shapley value provided a very parsimonious description for the observed data.

We also added this clarification to the end of the Results section:

This suggests that the observed assembly process is consistent with the recursive logic of the fair assembly rule: the improved predictive performance is not driven by simply feeding in more data, but by adjusting the initial condition of the recursion based on observed, as opposed to fair, subcommunity outcomes.

Additional comments:

L145: Does this property of linearity even apply when means are taken across growth conditions (i.e., control vs antibiotics)?

Thank you, this is a good question. The property of linearity applies when the same coalition is subject to different games, which in our case represent different growth conditions. So, indeed, the sum of the Shapley values across the environments is also a Shapley value of the entire game across four different environments and formally justifies our approach of averaging over different games. However, in some cases the same species can be present in many communities. In this case, the property of linearity does not strictly speaking apply when averaging the total Shapley values across all communities per species. Intuitively, the species fair proportion thus partly depends on the community context. However, we believe that the ‘average Shapley value’ of a species is still indicative of the species overall performance.

We have now clarified this mathematical aspect in the main text:

We then turned to analyze the roles of the individual species by collecting their Shapley values from all the communities they were present. Note that by the property of linearity, averaging the Shapley values of any given coalition across different environments constitutes a Shapley value of the entire game across environments. However, in our experiment, some species can belong to multiple different coalitions, and averaging over them is not anymore formally a Shapley value. Nevertheless, by averaging over all the individual Shapley values per species, the obtained mean Shapley value gives a measure of the species overall deserved share of the games’ output (performance). We show that the mean Shapley value of each species is correlated quite strongly with the true abundances (r = 0.754) but note that some species receive on average less than what their objective contribution to the community output is, while others gain a larger share than what could be considered fair (Fig. 2D).

Figure 2D: How many samples are averaged to get each point in this plot? Error bars would be helpful (along the x and y axes) and perhaps the size of the points should be scaled by the number of samples they are based on? Does averaging across the different conditions cause a

sort of regression towards the 1:1 line? Does Figure 4A contain the individual data points that are averaged to produce Figure 2D?

In Figure 2D we average the Shapley values per species across all environments and communities. The number of data points is not constant because some species may be present in multiple communities, while others only in one. The individual data points are in principle visible in Figure 4A (and also Figure 2A together when augmented with the species identities). In our view, error bars are not helpful here, because the Shapley values can significantly change in different environments. Nevertheless, we agree that showing that variability is helpful and have now added a new supplementary figure Fig. S1, which shows the Figure 2D across different environments. Based on this figure, we find that although averaging over the environments can introduce a sort of regression to the mean, most data points are nevertheless consistently in either side of the diagonal.

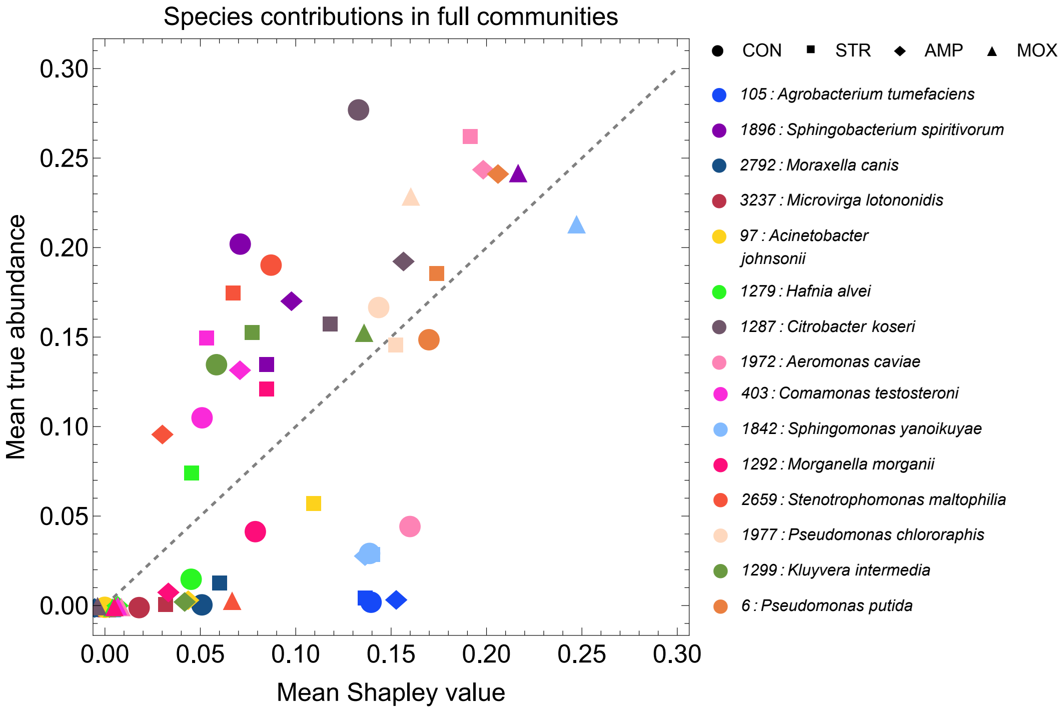

L176: Would you be able to explain in a bit more detail the role of the biculture scaling and how this relates to antagonism/synergism?

If species are synergetic, this means that the biculture yield is higher than either of the monoculture yields (and vice versa for antagonism). Because the biculture yield appears in the denominator, it thus scales the monoculture difference. For example, assume that Y1=1 and Y2=1.5, so species to 2 is 50 % more efficient. If Y12=1.5, then the fair proportions would be 1/3 and 2/3. Now, if the biculture yield would be higher, the fair proportions would be more equal (e.g. if Y12=2, then the fair proportions would be 3/8 and 5/8). On the other hand, if Y12=1, then the fair proportions would be even more unequal, 1/4 and 3/4. Thus, larger the total output, larger the fair proportion not only in absolute, but also relative terms. In the limit where the biculture yield is very large, the species' fair proportions would tend to equal proportions.

We clarified what we mean by antagonism and synergy also in the text:

This uniquely defines the fair distribution in case of two species for any ecological dynamics, assigning a higher fair proportion for the species with more efficient resource conversion efficiency while the biculture value scales the monoculture difference more strictly in case of antagonism (lower biculture yield) and more loosely in case of synergy (higher biculture yield) allowing for more equal proportions. The fair distribution therefore has the interesting feature that as the total output increases, the fair proportion of the less abundant species increases not only in absolute, but also in relative terms.

L176: I was wondering if the ideas of "selection effects" and "complementarity effects" from the Biodiversity-Ecosystem Functioning literature (e.g., https://doi.org/10.1038/35083573) are related to Shapley values in any way? Complementarity effects seem like a sort of synergism where groups do better than the sum of subgroups, and selection effects might produce "unfair" communities?

Thank you for pointing out this. While we agree that there is some overlap, there is no direct mapping between unfairness and selection effects because high relative abundances can also be fair or even driven by positive interactions. We could not think of a way that would allow us to consistently relate these concepts and therefore think making this connection might appear as potentially confusing. Rather, synergism in general is relevant here because the entire concept of Shapley value is originally based on the idea of cooperating coalitions.

L305: Is the observed lag a true biological lag (e.g., due to phenotypic/metabolic switching), or is it a limitation of using OD as a proxy for abundance, where growth below the detection limit might cause an artificial lag?

We acknowledge that there could be growth below the detection limit of the instruments used. However, we believe that the observed lag is not an experimental artefact, but indicative of slower onset of growth. In other words, if there is growth, eventually it surpasses the detection limit, and the longer it takes to be detectable indicates actual slower onset of biological growth. Also, because we are comparing the relative lags obtained with the same protocol, it provides an unbiased way to quantify the early growth across the species.

L337: it is certainly a big limitation of this approach that each combination of species must be observed. However, only requiring total yield rather than relative abundances for these subgroups is a big advantage - especially for systems where species identification is a challenge (e.g., microbial systems, zooplankton). I wonder if this advantage may be less noticeable in macro-systems, where measuring the total yield of say plants or seashells may be just as easy as estimating relative abundances?

We cannot really comment on how easy it is to measure biomass compared to relative abundances in other systems of interest, but at least for microbial systems this is a major advantage. Certainly, both are in some sense fundamental objects of interests across different

ecosystems, which in principle makes the introduced framework broadly applicable. In our view, the Shapley value framework can be useful also in other ways besides avoiding the need to estimate relative abundances.

L394: a single batch growth cycle makes a lot of sense here where biomass is the "currency" in a game of growth, but presumably all of the fairness logic can be directly applied to systems where resources are supplied in other ways (e.g., pulsed as in serial transfer experiments or continuous as in chemostat experiments)?

Yes, the Shapley value analysis and the fairness logic can be used analogously in any context of growth also in other experimental protocols. The Shapley value analysis can further be used identically for any data-generating mechanism and to any community output or function of interest (which would then define a new game). However, the fairness element may not be sensible for all games or how to measure the empirical distribution may vary depending on the function of interest.

Reviewer #2:

The paper takes a well developed concept from cooperative game theory and applies it to data from experimental evolution with microbes. According to my knowledge, this is a novel - and in my opinion ingenious - approach to get additional insights from abundance data and to consider the transitions in communities with an added (or subtracted) new type. The paper is well written - when I read it, I had many questions, but usually found them answered a few lines further down. I am not sure if this approach will become a new gold standard to look into the data, but the paper is taking an approach that I have never seen before and I think it will be very interesting to a wide readership, including microbiologists, theoretical ecologists and scientists related areas.

Nonetheless, I have a few comments:

We'd like to thank Reviewer 2 for the nice and encouraging comments towards our work. Below we reply to the raised comments and observations case-by-case.

- The paper considers cooperative game theory rather than competitive game theory, which is often used in evolution. While the authors make this point very clear, I think the paper would benefit from a short paragraph discussing both approaches in more detail, i.e. I think it would be good to expand a bit on line 61-63.

Thank you, we have now added more introductory discussion as suggested:

The field of game theory can be roughly divided to non-cooperative game theory, which focuses on modelling individual strategic behavior in a competitive setting, and cooperative game theory, which in contrast focuses solely on the outputs of different coalitions of players and how the total output is distributed among the players (either by bargaining or fair redistribution). While game

theory has been extensively applied, and indeed also developed, within the broader fields of evolution and ecology (added reference), existing eco-evolutionary theory is predominantly making use of the concepts of non-cooperative game theory by attempting to model microscopic interactions and strategy dynamics either at the level of individuals, species, or genes as players. However, measuring these underlying mechanistic interactions can be generally very difficult in case of complex biological systems even for simple minimal models, such as the generalized Lotka-Volterra model commonly employed for studying ecological communities (27, 28). Here we demonstrate, perhaps counterintuitively, that concepts of cooperative game theory can be meaningfully applied also in an inherently non-cooperative biological setting, where competition and exploitation are the norm (29, 30). As we will show, this opens up new, top-down methods to model and predict assembly of complex ecological communities.

We introduce the ecological Game of Growth where we first measure the biomass growth – the universal currency of living systems – in different species assemblages and then analyze the game's distributional properties using the concept of Shapley value (see Fig. 1).

- Along similar lines, evolutionary game theory of matrix games in terms of the replicator dynamics is dynamically equivalent to the Lotka-Volterra equations (see the book of Hofbauer & Sigmund), but the underlying conceptual ideas are very different: An n x n game corresponds to an ecological system with n-1 types which have different growth rates. Only if all growth rates are identical, we can speak of a game that has direct correspondence in ecology. Therefore, the term "game of growth" is a bit confusing for somebody like me, who looks at this from an evolutionary game theory perspective. Maybe the term "game of growth" can be made a bit clearer or even avoided? However, this issue could potentially already be resolved by expanding on line 61-63.

Our current approach here is purely an ecological game, the game of consuming and producing resources that are then converted to biomass, and there is no evolution. While we think adopting an evolutionary perspective on fairness is a fascinating topic for future study, evolutionary game theory does not really enter the present discussion of the 'game' defined here so we don't see the term confusing. We hope that the modifications above help to resolve any potential for confusion also in this part.

- Maybe I missed it, but how where the bacterial species selected? Is there a basis for these communities? To me, this is far from essential, but it would be good to know how the set of 16 species emerged.

We provide the selection procedure in detail in the Methods section and now refer to it also in the main text. We also added the following information to the Methods as well as moved the Table S1 to the methods:

The strains chosen into the species pool were a subset of species based on prior research (see Table 1 and ref. 44) and they were known to be able to coexist even for extended periods of time, with their antibiotic resistance profiles well-characterized.

In summary, I strongly recommend its publication - it has the potential to start a new research area.

Thank you, we appreciate the insights and enthusiasm.
