# Supplementary material for: Normative assembly rule reveals fairness in microbial communities
Source: PLoS Biol. 2026 Jun 24;24(6):e3003872. doi: 10.1371/journal.pbio.3003872 (PMC13327520; doi:10.1371/journal.pbio.3003872)
Supplement: Peer review history

# Editor report: Decision Letter 0

**DOI:** 10.1371/journal.pbio.3003872.r001

**Authors:** Roland G Roberts

11 Feb 2026

Dear Dr Kuosmanen,

Thank you for submitting your manuscript entitled "Normative assembly rule reveals fairness in microbial communities" for consideration as a Short Reports by PLOS Biology.

Your manuscript has now been evaluated by the PLOS Biology editorial staff, as well as by an academic editor with relevant expertise, and I'm writing to let you know that we would like to send your submission out for external peer review. Please accept my apologies for the delay incurred while we sought external expert advice.

However, before we can send your manuscript to reviewers, we need you to complete your submission by providing the metadata that is required for full assessment. To this end, please login to Editorial Manager where you will find the paper in the 'Submissions Needing Revisions' folder on your homepage. Please click 'Revise Submission' from the Action Links and complete all additional questions in the submission questionnaire.

Once your full submission is complete, your paper will undergo a series of checks in preparation for peer review. After your manuscript has passed the checks it will be sent out for review. To provide the metadata for your submission, please Login to Editorial Manager (https://www.editorialmanager.com/pbiology) within two working days, i.e. by Feb 13 2026 11:59PM.

If your manuscript has been previously peer-reviewed at another journal, PLOS Biology is willing to work with those reviews in order to avoid re-starting the process. Submission of the previous reviews is entirely optional and our ability to use them effectively will depend on the willingness of the previous journal to confirm the content of the reports and share the reviewer identities. Please note that we reserve the right to invite additional reviewers if we consider that additional/independent reviewers are needed, although we aim to avoid this as far as possible. In our experience, working with previous reviews does save time.

If you would like us to consider previous reviewer reports, please edit your cover letter to let us know and include the name of the journal where the work was previously considered and the manuscript ID it was given. In addition, please upload a response to the reviews as a 'Prior Peer Review' file type, which should include the reports in full and a point-by-point reply detailing how you have or plan to address the reviewers' concerns.

During the process of completing your manuscript submission, you will be invited to opt-in to posting your pre-review manuscript as a bioRxiv preprint. Visit http://journals.plos.org/plosbiology/s/preprints for full details. If you consent to posting your current manuscript as a preprint, please upload a single Preprint PDF.

Feel free to email us at plosbiology@plos.org if you have any queries relating to your submission.

Kind regards,

Roli Roberts

Roland Roberts, PhD

Senior Editor

PLOS Biology

rroberts@plos.org

# Peer review: Decision Letter 1

**DOI:** 10.1371/journal.pbio.3003872.r002

**Authors:** Roland G Roberts

26 Mar 2026

Dear Dr Kuosmanen,

Thank you for your patience while your manuscript "Normative assembly rule reveals fairness in microbial communities" went through peer-review at PLOS Biology. Your manuscript has now been evaluated by the PLOS Biology editors, an Academic Editor with relevant expertise, and by two independent reviewers.

You'll see that reviewer #1 is very positive about the study overall, but wonders if you could explore mechanistic aspects of the causal inference part of the paper (which s/he sees as more convincing) and expresses scepticism about the predictive value of the approach, which seems to perform poorly. Reviewer #2 is also positive; while s/he’s not convinced that the approach will be widely used, thay think that it is provocative and will trigger further work; s/he has some minor textual requests.

IMPORTANT: I discussed the points made by the reviewers with the Academic Editor, who asked me to suggest to you that taking on board rev 1's main suggestions is likely to increase the impact of the work and would make it clear to readers where the real strength and novelty lies.

In light of the reviews, which you will find at the end of this email, we are pleased to offer you the opportunity to address the comments from the reviewers in a revision that we anticipate should not take you very long. We will then assess your revised manuscript and your response to the reviewers' comments with our Academic Editor aiming to avoid further rounds of peer-review, although we might need to consult with the reviewers, depending on the nature of the revisions.

In addition to these revisions, you may need to complete some formatting changes, which you will receive in a follow up email. A member of our team will be in touch with a set of requests shortly. If you do not receive a separate email within a few days, please assume that checks have been completed, and no additional changes are required.

We expect to receive your revised manuscript within 1 month. Please email us (plosbiology@plos.org) if you have any questions or concerns, or would like to request an extension.

At this stage, your manuscript remains formally under active consideration at our journal; please notify us by email if you do not intend to submit a revision so that we withdraw the manuscript.

**IMPORTANT - SUBMITTING YOUR REVISION**

Your revisions should address the specific points made by each reviewer. Please submit the following files along with your revised manuscript:

1. A 'Response to Reviewers' file - this should detail your responses to the editorial requests, present a point-by-point response to all of the reviewers' comments, and indicate the changes made to the manuscript.

*NOTE: In your point-by-point response to the reviewers, please provide the full context of each review. Do not selectively quote paragraphs or sentences to reply to. The entire set of reviewer comments should be present in full and each specific point should be responded to individually.

You should also cite any additional relevant literature that has been published since the original submission and mention any additional citations in your response.

2. In addition to a clean copy of the manuscript, please also upload a 'track-changes' version of your manuscript that specifies the edits made. This should be uploaded as a "Revised Article with Changes Highlighted " file type.

*Resubmission Checklist*

When you are ready to resubmit your revised manuscript, please refer to this resubmission checklist: https://plos.io/Biology_Checklist

To submit a revised version of your manuscript, please go to https://www.editorialmanager.com/pbiology/ and log in as an Author. Click the link labelled 'Submissions Needing Revision' where you will find your submission record.

Please make sure to read the following important policies and guidelines while preparing your revision:

*Published Peer Review*

Please note while forming your response, if your article is accepted, you may have the opportunity to make the peer review history publicly available. The record will include editor decision letters (with reviews) and your responses to reviewer comments. If eligible, we will contact you to opt in or out. Please see here for more details:

https://blogs.plos.org/plos/2019/05/plos-journals-now-open-for-published-peer-review/

*PLOS Data Policy*

Please note that as a condition of publication PLOS' data policy (http://journals.plos.org/plosbiology/s/data-availability) requires that you make available all data used to draw the conclusions arrived at in your manuscript. If you have not already done so, you must include any data used in your manuscript either in appropriate repositories, within the body of the manuscript, or as supporting information (N.B. this includes any numerical values that were used to generate graphs, histograms etc.). For an example see here: http://www.plosbiology.org/article/info%3Adoi%2F10.1371%2Fjournal.pbio.1001908#s5

*Protocols deposition*

To enhance the reproducibility of your results, we recommend that if applicable you deposit your laboratory protocols in protocols.io, where a protocol can be assigned its own identifier (DOI) such that it can be cited independently in the future. Additionally, PLOS ONE offers an option for publishing peer-reviewed Lab Protocol articles, which describe protocols hosted on protocols.io. Read more information on sharing protocols at https://plos.org/protocols?utm_medium=editorial-email&utm_source=authorletters&utm_campaign=protocols

Thank you again for your submission to our journal. We hope that our editorial process has been constructive thus far, and we welcome your feedback at any time. Please don't hesitate to contact us if you have any questions or comments.

Sincerely,

Roli Roberts

Roland Roberts, PhD

Senior Editor

PLOS Biology

rroberts@plos.org

----------------------------------------------------------------

REVIEWERS' COMMENTS:

Reviewer #1:

The manuscript "Normative assembly rule reveals fairness in microbial communities" introduces the idea of Shapley values to community ecologists and uses microbial growth assays as a first case study. The work is presented clearly, the limitations (e.g., need to assemble all possible combinations of species) are explicitly addressed, and exciting future directions are proposed. It is an excellent example of the value of cross-disciplinary research, and microbial systems are the perfect starting point for introducing these ideas to ecology. I was not familiar with the concept of Shapley values before, and this manuscript made me think deeply about the trade-offs between the mechanistic and phenomenological approaches ecologists take when modelling community assembly.

Based on the paragraph starting at L322, the authors present the two key applications of their work as: (i) a new model-agnostic framework for making causal inferences about species composition and interactions, and (ii) a new approach for predicting community assembly outcomes. I was very interested by the first application, but I was a little less convinced by the second.

(i) I was intrigued by how this perspective of fairness could provide new insights into the biology of community assembly. Unravelling the mechanisms that make communities fair or unfair seems like an exciting line of research. Figure S1 and the related discussion of lag time really sparked my interest as it felt intuitive how increased lag time may prevent a species from being able to use up its fair share of resources. However, it seems that increased lag time can cause a species to be both above and below the 1:1 line in Figure 2D; lags can be advantageous or disadvantageous depending on the context. How could this be happening? Another striking result, where abundant species seem to always gain more biomass than their fair share (L133, Figure 2, and the hour-glass pattern in Figure 4) also intrigued me, but I thought the underlying biological mechanism could be explored a bit more. Once a species has an advantage it somehow is able to make the most of it? Are the authors confident that this result isn't a bias associated with how the sequencing data is converted to relative frequencies? Finally, the tentative conclusion arising from Figure 3E - that fairness seems to set an upper limit to evenness - felt like it deserved much more mechanistic discussion. In general, I thought the authors could lean more into the causal inferences/biological mechanisms revealed by their fairness approach, so that it was clearer how this new way of analysing ecological data can provide new insights.

(ii) The second application of the framework - predicting community outcomes - was a bit less compelling in my opinion. Apologies if I am misunderstanding the analyses, but to me it seemed that the predictions based purely on Shapley values are shown in Figure 4A. These predictions require knowing the total yield of every combination of species, but their accuracy seems very poor considering how much data they are based on. If prediction is the goal, I doubt that the fair distribution is a good choice (the authors do rightly present it as more of a sort of null model). Figure 4B and 4C look better (still not great though given that the predicted frequencies can still be off by as much as ~0.4), but they are augmented with even more data: relative frequencies for all pairs and all triplets. Once the frequency of every species in every subcommunity can be used in the prediction (as in Figure 4C), then presumably the fair assembly rule will be outperformed by even simple statistical models, and perhaps even by minimal ecological models like a generalized Lotka-Volterra model with higher-order interactions? Unless the authors can show that their fairness framework provides better predictions than current approaches, maybe it would be better to focus more on the causal inference side of the work?

Additional comments:

L145: Does this property of linearity even apply when means are taken across growth conditions (i.e., control vs antibiotics)?

Figure 2D: How many samples are averaged to get each point in this plot? Error bars would be helpful (along the x and y axes) and perhaps the size of the points should be scaled by the number of samples they are based on? Does averaging across the different conditions cause a sort of regression towards the 1:1 line? Does Figure 4A contain the individual data points that are averaged to produce Figure 2D?

L176: Would you be able to explain in a bit more detail the role of the biculture scaling and how this relates to antagonism/synergism?

L176: I was wondering if the ideas of "selection effects" and "complementarity effects" from the Biodiversity-Ecosystem Functioning literature (e.g., https://doi.org/10.1038/35083573) are related to Shapley values in any way? Complementarity effects seem like a sort of synergism where groups do better than the sum of subgroups, and selection effects might produce "unfair" communities?

L305: Is the observed lag a true biological lag (e.g., due to phenotypic/metabolic switching), or is it a limitation of using OD as a proxy for abundance, where growth below the detection limit might cause an artificial lag?

L337: it is certainly a big limitation of this approach that each combination of species must be observed. However, only requiring total yield rather than relative abundances for these subgroups is a big advantage - especially for systems where species identification is a challenge (e.g., microbial systems, zooplankton). I wonder if this advantage may be less noticeable in macro-systems, where measuring the total yield of say plants or seashells may be just as easy as estimating relative abundances?

L394: a single batch growth cycle makes a lot of sense here where biomass is the "currency" in a game of growth, but presumably all of the fairness logic can be directly applied to systems where resources are supplied in other ways (e.g., pulsed as in serial transfer experiments or continuous as in chemostat experiments)?

Reviewer #2:

The paper takes a well developed concept from cooperative game theory and applies it to data from experimental evolution with microbes. According to my knowledge, this is a novel - and in my opinion ingenious - approach to get additional insights from abundance data and to consider the transitions in communities with an added (or subtracted) new type. The paper is well written - when I read it, I had many questions, but usually found them answered a few lines further down. I am not sure if this approach will become a new gold standard to look into the data, but the paper is taking an approach that I have never seen before and I think it will be very interesting to a wide readership, including microbiologists, theoretical ecologists and scientists related areas.

Nonetheless, I have a few comments:

-The paper considers cooperative game theory rather than competitive game theory, which is often used in evolution. While the authors make this point very clear, I think the paper would benefit from a short paragraph discussing both approaches in more detail, i.e. I think it would be good to expand a bit on line 61-63.

-Along similar lines, evolutionary game theory of matrix games in terms of the replicator dynamics is dynamically equivalent to the Lotka-Volterra equations (see the book of Hofbauer & Sigmund), but the underlying conceptual ideas are very different: An n x n game corresponds to an ecological system with n-1 types which have different growth rates. Only if all growth rates are identical, we can speak of a game that has direct correspondence in ecology. Therefore, the term "game of growth" is a bit confusing for somebody like me, who looks at this from an evolutionary game theory perspective. Maybe the term "game of growth" can be made a bit clearer or even avoided? However, this issue could potentially already be resolved by expanding on line 61-63.

-Maybe I missed it, but how where the bacterial species selected? Is there a basis for these communities? To me, this is far from essential, but it would be good to know how the set of 16 species emerged.

In summary, I strongly recommend its publication - it has the potential to start a new research area.

# Author comment: Author response to Decision Letter 2

**DOI:** 10.1371/journal.pbio.3003872.r003

24 Apr 2026

Attachment Submitted filename: Response-Letter.pdf

# Editor report: Decision Letter 2

**DOI:** 10.1371/journal.pbio.3003872.r004

**Authors:** Roland G Roberts

22 May 2026

Dear Dr Kuosmanen,

Thank you for your patience while we considered your revised manuscript "Normative assembly rule reveals fairness in microbial communities" for publication as a Short Report at PLOS Biology. This revised version of your manuscript has been evaluated by the PLOS Biology editors and the Academic Editor.

Based on our Academic Editor's assessment of your revision, we are likely to accept this manuscript for publication, provided you satisfactorily address the remaining points raised by the Academic Editor (see the foot of this email) and the following data and other policy-related requests.

IMPORTANT - please attend to the following:

a) Please address the remaining concerns raised by the Academic Editor (these mostly relate to points previously raised by reviewer #1, and your responses to them).

b) I note that you already have an associated OSF deposition (https://osf.io/a7yzp/overview) which contains five .csv data files, and one Mathematica .nb notebook. Please could you confirm whether the data and code in this deposition are sufficient to recreate the Figures?

c) Please cite the location of the data clearly in all relevant main and supplementary Figure legends, e.g. “The data and code needed to create this Figure can be found in https://osf.io/a7yzp/overview"

d) Please include the URLs of your funders in the Financial Disclosure statement.

As you address these items, please take this last chance to review your reference list to ensure that it is complete and correct. If you have cited papers that have been retracted, please include the rationale for doing so in the manuscript text, or remove these references and replace them with relevant current references. Any changes to the reference list should be mentioned in the cover letter that accompanies your revised manuscript.

In addition to these revisions, you may need to complete some formatting changes, which you will receive in a follow up email. A member of our team will be in touch with a set of requests shortly. If you do not receive a separate email within a few days, please assume that checks have been completed, and no additional changes are required.

We expect to receive your revised manuscript within two weeks.

To submit your revision, please go to https://www.editorialmanager.com/pbiology/ and log in as an Author. Click the link labelled 'Submissions Needing Revision' to find your submission record. Your revised submission must include the following:

- a cover letter that should detail your responses to any editorial requests, if applicable, and whether changes have been made to the reference list

- a Response to Reviewers file that provides a detailed response to the reviewers' comments (if applicable, if not applicable please do not delete your existing 'Response to Reviewers' file.)

- a track-changes file indicating any changes that you have made to the manuscript.

NOTE: If Supporting Information files are included with your article, note that these are not copyedited and will be published as they are submitted. Please ensure that these files are legible and of high quality (at least 300 dpi) in an easily accessible file format. For this reason, please be aware that any references listed in an SI file will not be indexed. For more information, see our Supporting Information guidelines:

https://journals.plos.org/plosbiology/s/supporting-information

*Published Peer Review History*

Please note that you may have the opportunity to make the peer review history publicly available. The record will include editor decision letters (with reviews) and your responses to reviewer comments. If eligible, we will contact you to opt in or out. Please see here for more details:

https://plos.org/published-peer-review-history/

*Press*

Should you, your institution's press office or the journal office choose to press release your paper, please ensure you have opted out of Early Article Posting on the submission form. We ask that you notify us as soon as possible if you or your institution is planning to press release the article.

*Protocols deposition*

To enhance the reproducibility of your results, we recommend that if applicable you deposit your laboratory protocols in protocols.io, where a protocol can be assigned its own identifier (DOI) such that it can be cited independently in the future. Additionally, PLOS ONE offers an option for publishing peer-reviewed Lab Protocol articles, which describe protocols hosted on protocols.io. Read more information on sharing protocols at https://plos.org/protocols?utm_medium=editorial-email&utm_source=authorletters&utm_campaign=protocols

Please do not hesitate to contact me should you have any questions.

Sincerely,

Roland

Roland Roberts, PhD

Senior Editor

rroberts@plos.org

PLOS Biology

------------------------------------------------------------------------

CODE POLICY

Per journal policy, if you have generated any custom code during the course of this investigation, please make it available without restrictions. Please ensure that the code is sufficiently well documented and reusable, and that your Data Statement in the Editorial Manager submission system accurately describes where your code can be found. More information on our Code Policy, what and how to share can be found here: https://journals.plos.org/plosbiology/s/code-availability

Please note that we cannot accept sole deposition of code in GitHub, as this could be changed after publication. However, you can archive this version of your publicly available GitHub code to Zenodo. Once you do this, it will generate a DOI number, which you will need to provide in the Data Accessibility Statement (you are welcome to also provide the GitHub access information). See the process for doing this here: https://docs.github.com/en/repositories/archiving-a-github-repository/referencing-and-citing-content

------------------------------------------------------------------------

DATA NOT SHOWN?

- Please note that per journal policy, we do not allow the mention of "data not shown", "personal communication", "manuscript in preparation" or other references to data that is not publicly available or contained within this manuscript. Please either remove mention of these data or provide figures presenting the results and the data underlying the figure(s).

------------------------------------------------------------------------

COMMENTS FROM THE ACADEMIC EDITOR:

Please revisit the following points raised by the reviewers in the previous round:

(1) Reviewer 1 "Another striking result, where abundant species seem to always gain more biomass than their fair share (L133, Figure 2, and the hour-glass pattern in Figure 4) ....." Response: "Yes, the general pattern that we observe (as in Fig. 2C) is that biomass is more concentrated to the most prevalent species compared to the fair distribution. However, it is worth to note that in Figure 2 analysis, these may or may not be the same species...." This seems like both a missed opportunity but also a major caveat of their finding. Is it the case that they can really say the dominant player took more than their fair share if it is more often than not the same species? This surely is a question of statistical rigor and it seems the authors can and should differentiate species identity from dominance if a key takeaway pattern is that dominant taxa take more than their fair share. Alternatively, the authors should make clear that this is a caveat/limitation of their results.

(2) Reviewer 1 takes issue with the focus on using the models to predict community assembly and the authors push back that prediction is not the goal. However, the abstract still states "Next, we develop a fair assembly rule based on the recursive definition of Shapley value that can predict also unfairly assembled community compositions. Our results give unique empirical insights into the distributive function of ecological dynamics and lay down the theoretical foundations of what might become a normative community assembly theory." which clearly emphasizes this as a move towards predictive community assembly. As such, I would be surprised in reviewer 1 found this a satisfying response. Perhaps the authors could spend some time in the discussion discussing the strengths and weaknesses of this approach in regard to its ability to predict community assembly? Or even better, in my opinion would be to reduce the emphasize on this approach for prediction of assembly as opposed to understanding composition.

# Author comment: Author response to Decision Letter 3

**DOI:** 10.1371/journal.pbio.3003872.r005

5 Jun 2026

Attachment Submitted filename: Response-Letter_auresp_3.pdf

# Editor report: Decision Letter 3

**DOI:** 10.1371/journal.pbio.3003872.r006

**Authors:** Roland G Roberts

8 Jun 2026

Dear Dr Kuosmanen,

Thank you for the submission of your revised Short Report "Normative assembly rule reveals fairness in microbial communities" for publication in PLOS Biology. On behalf of my colleagues and the Academic Editor, Britt Koskella, I'm pleased to say that we can in principle accept your manuscript for publication, provided you address any remaining formatting and reporting issues. These will be detailed in an email you should receive within 2-3 business days from our colleagues in the journal operations team; no action is required from you until then. Please note that we will not be able to formally accept your manuscript and schedule it for publication until you have completed any requested changes.

Please take a minute to log into Editorial Manager at http://www.editorialmanager.com/pbiology/, click the "Update My Information" link at the top of the page, and update your user information to ensure an efficient production process.

PRESS: We frequently collaborate with press offices. If your institution or institutions have a press office, please notify them about your upcoming paper at this point, to enable them to help maximise its impact. If the press office is planning to promote your findings, we would be grateful if they could coordinate with biologypress@plos.org. If you have previously opted in to the early version process, we ask that you notify us immediately of any press plans so that we may opt out on your behalf.

We also ask that you take this opportunity to read our Embargo Policy regarding the discussion, promotion and media coverage of work that is yet to be published by PLOS. As your manuscript is not yet published, it is bound by the conditions of our Embargo Policy. Please be aware that this policy is in place both to ensure that any press coverage of your article is fully substantiated and to provide a direct link between such coverage and the published work. For full details of our Embargo Policy, please visit http://www.plos.org/about/media-inquiries/embargo-policy/.

Thank you again for choosing PLOS Biology for publication and supporting Open Access publishing. We look forward to publishing your study.

Sincerely,

Roli Roberts

Roland G Roberts, PhD, PhD

Senior Editor

PLOS Biology

rroberts@plos.org
